# Substrate-decoupled, bulk-acoustic wave gyroscopes: Design and evaluation of next-generation environmentally robust devices

**DOI:** 10.1038/micronano.2016.15

**Published:** 2016-04-25

**Authors:** Diego E. Serrano, Mohammad F. Zaman, Amir Rahafrooz, Peter Hrudey, Ron Lipka, Duane Younkin, Shin Nagpal, Ijaz Jafri, Farrokh Ayazi

**Affiliations:** 1 Qualtré Inc., 225 Cedar Hill Street, Suite 112, Marlborough, MA 01752, USA; 2 Department of Electrical and Computer Engineering, Georgia Institute of Technology, 777 Atlantic Drive NW, Atlanta, GA 303332, USA

**Keywords:** Bulk-acoustic wave, environmental stability, MEMS gyroscope, support loss, vibration rejection

## Abstract

This paper reports on a new type of high-frequency mode-matched gyroscope with significantly reduced dependencies on environmental stimuli such as temperature, vibration, and shock. A novel stress-isolation system is used to effectively decouple an axis-symmetric bulk-acoustic wave (BAW) vibratory gyro from its substrate, minimizing the effect that external sources of error have on the offset and scale factor of the device. Substrate-decoupled (SD) BAW gyros with a resonance frequency of 4.3 MHz and *Q* values near 60 000 were implemented using the high aspect ratio poly and single-crystal silicon (HARPSS) process to achieve ultra-narrow capacitive gaps. Wafer-level packaged sensors were interfaced with a customized application-specific integrated circuit (ASIC) to achieve low variations in the offset across temperature (±26° s^−1^ from −40 to 85 °C), supreme random-vibration immunity (0.012° s^−1^ g_RMS_^−1^) and excellent shock rejection. With a scale factor of 800 μV (°s^−1^)^−1^, the SD-BAW gyro system attains a large full-scale range (±1250° s^−1^) with a non-linearity of less than 0.07%. A measured angle-random walk (ARW) of 0.39°/√h and a bias instability of 10.5°h^−1^ are dominated by the thermal and flicker noise of the integrated circuit (IC), respectively. Additional measurements using external electronics show bias-instability values as low as 3.5°h^−1^, which are limited by feed-through signals coupled from the drive loop to the sense channel, which can be further reduced through proper re-routing of the gyroscope pin-out configuration.

## Introduction

Micromachined gyroscopes have enabled a myriad of applications that range from basic motion detection for gaming to safety control systems in automobiles^1^. More recently, an increased interest in the use of microelectromechanical system inertial sensors for dead reckoning and pedestrian navigation in hand-held electronics has placed stringent requirements on the die size, power consumption, and overall performance of this type of device. To date, most commercially available rate sensors have been designed as low-frequency flexural tuning-fork gyroscopes (TFGs), which are typically sensitive to random vibrations and prone to linear accelerations, such as those experienced under shock. These limitations complicate the use of TFG technology in large-volume, high-end applications, particularly in personal navigation, for which dependencies on fluctuations in the environment translate into long-term drift in the output of the system. Additionally, in recent months, concerns about the high sensitivity of consumer-grade gyroscopes in response to low-frequency pressure signals that can be used to recover audio have increased as a potential threat for eavesdropping^2^, justifying the need for more environmentally robust rotation sensors.

Acceleration suppression mechanisms can be implemented in TFGs to alleviate part of this problem using redundant proof masses that reject shock and vibration as common-mode signals^3^. However, this compensation technique results in a significant increase in the size of these devices and could require electromechanical calibration to compensate for fabrication imperfections^4^, making them more suitable in low-volume applications.

As an alternative, the degenerate modes of bulk-acoustic wave (BAW) resonators can be used to implement axis-symmetric mode-matched gyroscopes that operate in the MHz range with high quality factors at moderate vacuum levels (that is, 1 to 10 Torr)^5^. Given their high-frequency nature, BAW gyros inherently reject the effects of random vibrations in the environment and are highly immune to shock.

However, like in any other type of gyroscope, differences in the loss mechanisms of the two degenerate modes can lead to damping coupling, which results in unwanted environmentally dependent offset variations^6^. In axis-symmetric gyros, fabrication or material imperfections can cause different support-loss rates for each of the modes, particularly if the devices are implemented in anisotropic substrates such as (100) single-crystal silicon (SCS). Therefore, to minimize the effects of damping mismatch, this paper presents a high-frequency gyro for *z*-axis rate detection that combines the properties of a BAW sensor with an isolation structure that significantly reduces anchor loss in the system. The substrate-decoupled (SD) BAW gyro thus attains a markedly improved environmental performance and offers the versatility necessary for high-volume production for consumer, automotive and industrial applications.

## Design and simulation

**Figure 1:**
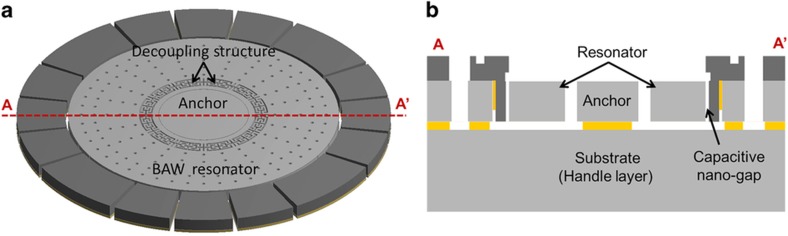
(**a**) Schematic view of the SD-BAW gyroscope. An active resonating gyro region is effectively decoupled from the substrate by a stress decoupling structure. (**b**) Electrodes with ultra-narrow capacitive gaps (270 nm) created with the HARPSS process are used for electrostatic actuation and readout. HARPSS, high aspect-ratio poly and single-crystal silicon; BAW, bulk-acoustic wave; SD, substrate-decoupled.

A schematic diagram of the SD-BAW gyroscope is shown in Figure 1. The sensor consists of an active resonator region anchored through a stress isolation system, which is designed to effectively decouple the gyro from its substrate at the resonance frequency of the modes of vibration. Electrodes with ultra-narrow capacitive gaps (270 nm) surround the structure to allow electrostatic excitation, readout, and frequency tuning^7^.

### Basic principles of operation

Similar to ring- and shell-type gyroscopes^8^, SD-BAW gyros are axis-symmetric devices that use the degenerate modes of a vibrating structure to detect rotation^5^. Therefore, the behavior of an SD-BAW device is described by two orthogonal, second-order systems coupled to each other by a Coriolis force that is proportional to an applied angular velocity:

(1a)m11q¨1(t)+b11q˙1(t)+b12q˙2(t)+k11q1(t)+k12q2(t)=∑i=1kF1,i−2λm22Ω(t)q˙2(t)

(1b)m22q¨2(t)+b22q˙2(t)+b21q˙1(t)+k22q2(t)+k21q1(t)=∑i=1kF2,i+2λm11Ω(t)q˙1(t)

In Equations (1a) and (1b), *Ω*(*t*) corresponds to the rate of rotation applied around an axis normal to the plane of modal vibration. The factor *λ* is known as the angular gain of the gyroscope, which is dictated by the Bryan effect^9^; its value is determined by the device’s geometry and mode shape. The terms *m*_11_, *k*_11_, and *b*_11_ are the effective mass, effective stiffness, and damping associated with mode 1, respectively; *m*_22_, *k*_22_, and *b*_22_ correspond to these same parameters of mode 2. In a perfect axis-symmetric gyroscope, the properties of both modes are identical, making *m*_11_=*m*_22_, *b*_11_=*b*_22_, *k*_11_=*k*_22_, and thus their resonances frequencies equal (*ω*_01_*=ω*_02_). However, material and fabrication imperfections lead to differences in the elastic and/or inertial characteristics of the degenerate modes, resulting a frequency split between them. Additionally, in an ideal vibratory gyro, the two modes should only be coupled to each other by the Coriolis forces −2*m*_22_*λ*$\dot{q}$_2_(*t*)*Ω*(*t*) and +2*m*_11_*λ*$\dot{q}$_*1*_(*t*)*Ω*(*t*); however, structural imperfections in a real device produce undesired mode-to-mode coupling^10^. These effects are captured in Equations (1a) and (1b) via the stiffness-coupling terms *k*_12_ and *k*_21_ and the damping-coupling coefficients *b*_12_ and *b*_21_.

The variables *F*_1,*i*_ and *F*_2,*j*_ correspond to the *k* and *l* electrostatic forces acting on modes 1 and 2, respectively, which are used to drive and control the mode of operation of the gyroscope and to compensate for the aforementioned elastic and inertial imperfections^11^.

**Figure 2:**
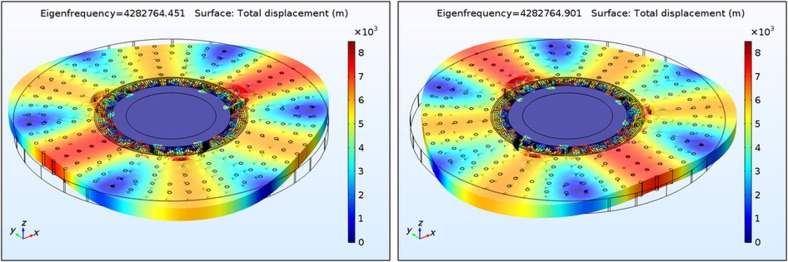
Modal simulation response of the second elliptical (*n*=3) in-plane modes of an SD-BAW gyroscope with 420 μm radius, created on a 40-μm-thick (100) SCS substrate (electrodes not shown; total displacement normalized with respect to mass matrix). BAW, bulk-acoustic wave; SCS, single-crystal silicon; SD, substrate-decoupled.

Lastly, the parameters *q*_1_(*t*) and *q*_2_(*t*) in Equations (1a) and (1b) are the generalized coordinates of modes 1 and 2, respectively, that determine the gyroscope’s modal displacements in combination with the mode-shape functions^12^. Figure 2 shows the simulated modal deformations of the second-elliptical (*n*=3) in-plane degenerate modes of an SD-BAW gyro that was created in a 40-μm-thick (100) SCS silicon-on-insulator (SOI) wafer with a radius of 420 μm. (100) SCS substrates were selected instead of (111) SCS substrates because the *n*=3 modes maintain degeneracy, and their resonance frequencies are less sensitive to crystalline misalignment^13^.

When configured as a rotation-rate sensor, one of the two degenerate modes of the gyroscope (that is, the drive mode) is excited into self-oscillation to establish a well-regulated displacement reference (that is, *q*_1_(*t*)=$\bar{q}$_1_sin(*ω*_drive_*t*), where $\bar{q}$_1_ is constant, and *ω*_drive_=*ω*_01_ ideally). In the presence of an angular velocity *Ω*(*t*), the second mode (that is, the sense mode) is excited by the generated Coriolis force, causing a displacement that can be read in the form of a current through a capacitive electrode aligned with the antinodes of the sense mode. If the frequency of the established drive-mode oscillation is equal to the resonance frequency of the sense mode (that is, *ω*_drive_=*ω*_02_), the generated displacement due to an applied rotation rate can be described by

(2)q2corilis(t)=2λQ2ω02Ω(t)q¯1sin(ω02t)

where *Q*_2_=*b*_22_/√(*k*_22_*m*_22_) is the quality factor of the sense mode. Therefore, *q*_2coriolis_(*t*) is an amplitude-modulated signal with an envelope proportional to the rate of rotation *Ω*(*t*) and carrier frequency and phase equal to those of the drive-loop displacement.

### Compensation of anisoinertia and anisoelasticity

Material and fabrication imperfections typically introduce differences in the masses (anisoinertia) and stiffnesses (anisoelasticity) of the two degenerate modes^10^. These effects translate into a frequency split that affects the overall performance of the gyro^14^. Additionally, these non-idealities tend to generate mode-to-mode coupling terms (*k*_12_ and *k*_21_) that translate into a sense-mode zero-rate output (ZRO) displacement error signal in an angular-rate sensor:

(3)q2stiffness(t)=Q2m22ω022k21q¯1cos(ω02t)

Although *q*_2stiffness_(*t*) is detrimental to gyro performance, the fact that its phase is 90° with respect to *q*_2coriolis_(*t*) makes it easy to identify and correct, particularly when using coherent synchronous demodulation^15^.

To compensate for this frequency split and the ZRO errors caused by the described anisoelasticity and anisoinertia, electrostatic spring softening can be used. Electrodes aligned with the antinodes of the degenerate modes of interest will introduce a direct stiffness term. Conversely, electrodes located between anti-nodes will affect the overall indirect stiffness coupling, allowing quadrature cancellation^10^.

**Figure 3:**
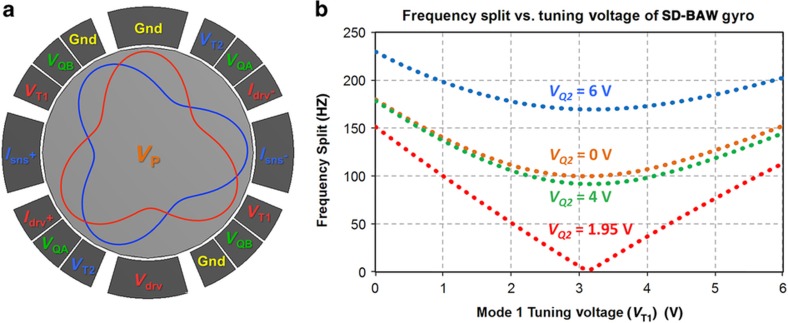
(**a**) Electrode configuration for the SD-BAW gyroscope. (**b**) Simulated electrostatic frequency-tuning and stiffness-decoupling for an imperfect (ellipsoidal) SD-BAW gyroscope. Mode decoupling was achieved with *V*_Q2_=1.95 V, and mode matching was achieved at *V*_T1_=3.15 V. The resonator was biased at *V*_P_=18 V with all other electrodes at a common-mode voltage of 2.5 V. The residual split was less than 0.05 Hz using a voltage resolution of 10 mV. BAW, bulk-acoustic wave; SD, substrate-decoupled.

Figure 3a shows the electrode configuration used for the SD-BAW gyroscope presented in this study. Electrodes *V*_T1_ and *V*_T2_ are used to tune the resonances of modes 1 and 2, respectively, to compensate for the frequency splits. Electrodes *V*_QA_ and *V*_QB_ are used to cancel out the mode-coupling terms. The pads labeled *I*_drv_+, *I*_drv_−, *I*_sns_+, *I*_sns_− correspond to the positive and negative readout electrodes for the drive and sense modes, and *V*_drv_ corresponds to the drive excitation electrode. The resonating gyro structure is biased at a polarization voltage *V*_P_; the electrodes labeled ‘Gnd’ are inactive in this study but can be used for additional frequency tuning, signal readout and/or modal excitation.

To verify the effectiveness of the tuning and decoupling electrodes, electromechanical FEA simulations were performed on an imperfect SD-BAW gyroscope. In the model, the outer boundary of the resonator was built as an ellipse rather than a perfect circle to capture the effects of lens astigmatism that can be produced during fabrication in the lithography step that defines the structure. The difference between the ellipse semi-axes was obtained empirically from previous generations of BAW resonators. Figure 3b shows an example of the frequency-tuning and mode-decoupling response when *V*_T1_ and *V*_Q2_ are varied. Only when the modes are fully decoupled can a frequency split of 0 Hz be achieved^10^.

### Mechanical reduction of anisodamping

The damping terms *b*_11_ and *b*_22_ in Equations (1a) and (1b) are a measure of the amount of vibration energy lost by each of the two modes typically to the surrounding environment^16,17^. If the sources of damping are not aligned with the antinodes of the modes of interest, each mode will also experience a coupling force proportional to the vibration velocity of its degenerate pair^18^. This effect is captured by the terms *b*_12_ and *b*_21_, which will generate an undesired sense displacement ZRO in a rotation-rate sensor:

(4)q2damping(t)=−Q2m22ω02b21q¯1sin(ω02t)

By comparing Equations (2), (3), and (4), it can be concluded that unlike *q*_2stiffness_(*t*), the error signal *q*_2damping_(*t*) is in phase with *q*_2coriolis_(*t*), which makes them indistinguishable from each other^19^. Complex circuit architectures can be used to reject *q*_2damping_(*t*) as a common-mode signal using mode reversal^20^. However, in a rate gyroscope system, this approach results in either extremely large settling times to achieve alternating modal excitations (limiting the effective bandwidth of the system)^21^ or high power consumption to drive both modes simultaneously. Therefore, it is critical to guarantee that the damping mechanisms that affect the two modes are designed to be as symmetric as possible.

In a resonant system, the effects of damping are typically expressed in terms of its quality factor, which quantifies the ratio of the peak energy stored in the resonator *W* to the energy dissipated per cycle Δ*W*:

(5)Q=2πWΔW

The total value of *Q* is then determined by the contributions of all different sources of energy loss that affect the system, which include squeeze-film damping (SFD)^22^, thermoelastic damping (TED)^23^, support or anchor loss^24^, and other surface^25^ and intrinsic losses^16^, which are typically minimal in SD-BAW gyros. Given the high-frequency nature of SD-BAW gyros, SFD typically has minor contributions to the total *Q*, even at moderate pressure levels (for example, 1 to 10 Torr). Additionally, even when operated at higher pressures, the axis-symmetric nature of the gyro causes the SFD values of both modes to be identical, thus not contributing to damping coupling. Similarly, TED in SD-BAW gyros is determined by the localized compressive and extensive strain generated around the release holes in the resonator. The hole-pattern arrangement can then be designed to be axis-symmetric and affect both modes identically, minimizing the contribution of TED to the coupling terms.

Conversely, the value of the anchor loss of each of the two degenerate modes is highly dependent on how the resonator couples to its substrate. This occurs because, even though the maximum energy stored on each mode is a function of controllable parameters such as material properties and geometry, the energy dissipated per cycle depends on the integral of the strain energy density (SED) at the resonator−substrate interface^24^:

(6)ΔW=π∫VsσsϵsdV

**Figure 4:**
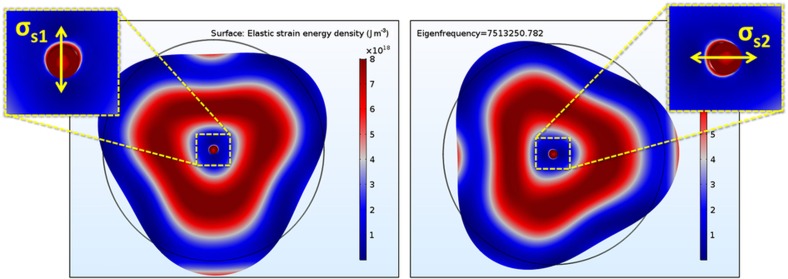
SED distribution of the second elliptical in-plane mode of a center-supported disk BAW resonator in (100) SCS. The anisotropic nature of the material causes a directionally dependent shear stress in the resonator−substrate boundary that is different for the two modes. (Red regions indicate where the SED is maximum; blue regions indicate where the SED is minimum.) BAW, bulk-acoustic wave; SCS, single-crystal silicon; SD, substrate-decoupled; SED, strain energy density.

In Equation (6), *Vs* is the volume of the support region, and σ_*s*_ and *ε*_*s*_ are the strain and stress at the resonator−substrate interface, respectively. A lower-bound ‘worst-case’ value of *Q* due to anchor loss (*Q*_anchor_) can be estimated for each of the modes by assuming that all the strain energy at the interface is dissipated into the substrate (that is, there are no acoustic reflections)^26,27^. However, this condition is only valid when the dimensions of the substrate are much larger than the wavelength of the acoustic wave generated by the resonator, which is rarely true. For example, the wavelength of the acoustic signal generated by a 4-MHz resonator traveling through silicon is approximately 2 mm, which would require the substrate to be much larger than this value in all directions to assume that no energy is reflected. Therefore, the real effective *Q*_anchor_ will be a function of how the energy propagates within the substrate, which can change dramatically in the presence of substrate spurious modes with frequencies that are strongly dependent on how the device is singulated, mounted, and attached to its surroundings. More importantly, the way the energy dissipates and reflects for each of the two degenerate modes of an axis-symmetric gyro can differ significantly, causing an excessive amount of damping coupling. For example, Figure 4 shows the SED distribution of the second-elliptical modes in a center-supported BAW disk resonator in (100) SCS. Due to the anisotropic nature of the device material, an effective shear stress is generated at the resonator/substrate interface that aligns with the antinode with the largest deformation of each of the modes. This will cause a loss mechanism that will propagate in different directions for each of the two modes and that depends on how the energy interacts with all spurious modes in the substrate.

Consequently, when a resonator is tightly coupled to its substrate, it would be difficult to estimate the effective anchor losses of each of the two modes; it would be even more complex to quantify their contribution to the undesired error signal *q*_2damping_(*t*).

**Figure 5:**
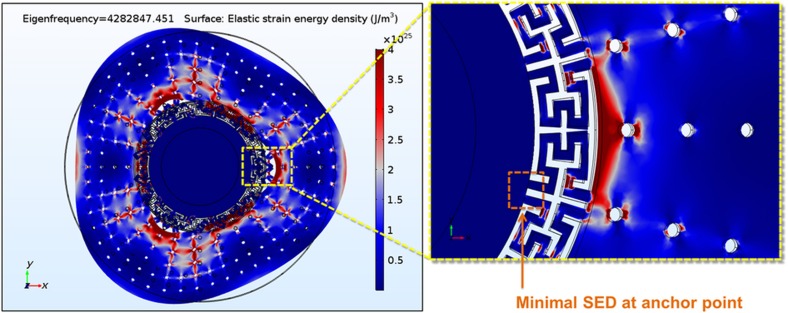
SED distribution for the second elliptical in-plane mode of the presented SD-BAW implemented in (100) SCS. The stress isolation system effectively attenuates the strain induced on the center support, minimizing anchor loss. BAW, bulk-acoustic wave; SCS, single-crystal silicon; SD, substrate-decoupled; SED, strain energy density.

A practical solution to this problem is to implement an effective acoustic isolation mechanism between the resonator structure and its surroundings. Several techniques to minimize anchor loss in microresonators have been reported in the literature, including the use of acoustic stop-band phononic crystal structures^28^, the addition of half-wavelength acoustic reflectors^29^, or the exploitation of the mismatch of material properties for acoustic isolation^30^. However, a less intricate way to isolate a resonant structure is including a stress isolation structure^31^. To reduce the final stress imparted to the anchoring point, the isolation apparatus should be capable of effectively attenuating the strain produced by the deformation in the resonator. Figure 5 shows a close-up view of the SED distribution in the stress isolation system used for an SD-BAW gyroscope. It is clearly shown that the decoupling structure attenuates the strain induced on the center anchor, providing excellent isolation between the resonator and its substrate.

Part of the energy that would be originally dissipated into the substrate will now be stored within the resonator; however, another portion of it will be dissipated in the form of heat generated within the new compressive and tensile regions in the decoupling system (that is, TED within the stress isolation structure). If the stress isolation components are arranged in an axis-symmetric fashion (that is, similar to the release holes), both modes will experience symmetric and equal losses, minimizing damping coupling.

To quantify the level of isolation provided by the added decoupling structure, a lower bound for the anchor loss of the SD-BAW gyro can be determined using FEA simulations with perfectly matched layers (PMLs)^26^. As previously discussed, this method does not provide an absolute anchor-loss value but does provide an indication of the level of decoupling between a resonant structure and its substrate. This limitation exists because only a small amount of energy can be transferred from the resonator into the substrate if the worst-case *Q*_anchor_ is extremely high; therefore, the effect of any reflections will also be small.

Table 1 summarizes the simulated results for the different losses of the second elliptical in-plane modes in both a center-supported BAW disk and an SD-BAW gyroscope with the same dimensions. It is shown that for the SD-BAW gyro *Q*_anchor_ is more than four orders of magnitude larger than the dominant loss mechanism in the system (*Q*_TED_), making its contributions to the *Q*_TOTAL_ small. Conversely, the *Q*_anchor_ of the center-support BAW disk is the dominant source of loss, making the overall system dependent on its substrate.

### Shock and vibration rejection

The shock and vibration performance of a gyroscope is determined by how well the design can reject acceleration signals. In TFGs, which typically have resonance frequencies within the spectrum of environmental shock and vibration stimuli, this is typically done by using redundant proof masses that respond differentially to a Coriolis force but produce a common-mode displacement that can be rejected during linear accelerations^32^. However, the effectiveness of this approach depends on how well design symmetry is maintained^14^ and on the level of modal cross-excitation generated by errors in the capacitive sidewalls^33^. Additionally, this technique fails to reject the acceleration response of other higher-order modes that can still be within the spectrum of interest.

**Figure 6:**
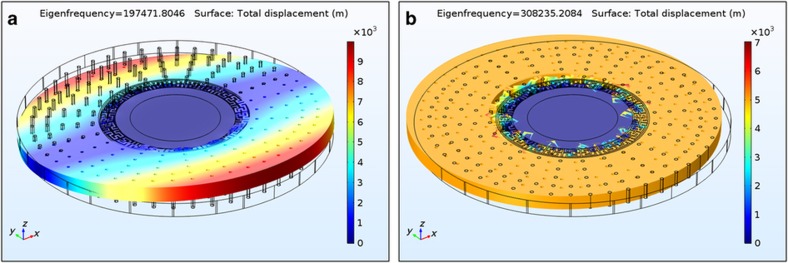
Simulation response for the two lowest resonance-frequency modes of an SD-BAW gyroscope. (**a**) The teeter−totter mode has a resonance frequency of 200 kHz, and (**b**) the in-plane translational mode has a resonance frequency of 300 kHz. BAW, bulk-acoustic wave; SD, substrate-decoupled.

Conversely, the high-frequency nature of SD-BAW gyroscopes makes them inherently robust to shock and vibration. The displacement experienced by any mechanical structure in the presence of an acceleration signal *a*(*t*) can be expressed in terms of the frequencies of the resonance modes with components aligned with the acceleration vector. Typically, the mode with the lowest frequency *ω*_0*a*_ will determine the majority of the displacement *q*_accel_(*t*)=1/*ω*_0*a*_^2^*a*(*t*). In a gyroscope, *q*_accel_(*t*) can modulate the sense mode displacement *q*_*2*_(*t*) if there is a residual ZRO signal due to stiffness or damping coupling, or cross-modal excitation in the case of TFGs. The acceleration-dependent ZRO can then be expressed as follows:

(7)q2accel(t)=κω0a2a(t)cos(α)sin(ω02t)

where *κ* is the normalized amplitude of the residual ZRO, and *α* is the angle between the direction of the applied acceleration and the sense-mode displacement. It is worth noting that the parameter *q*_accel_(*t*) will also modulate the carrier signal of *q*_2coriolis_(*t*) when the rotation rate is applied, even in the absence of ZRO. Therefore, reducing *κ* (that is, the ZRO amplitude) is important but not sufficient to create a gyro that is environmentally robust during operation; it is only through an increase in the value of *ω*_0*a*_ that *q*_2accel_(*t*) can be markedly reduced. Figure 6 shows the two lowest resonance modes of the SD-BAW gyroscope. With frequencies of 200 kHz (that is, teeter−totter mode) and 300 kHz (that is, in-plane translational mode), these two modes are well above the frequency spectrum environmental random vibration signals, which is typically below 50 kHz.

## Fabrication

**Figure 7:**
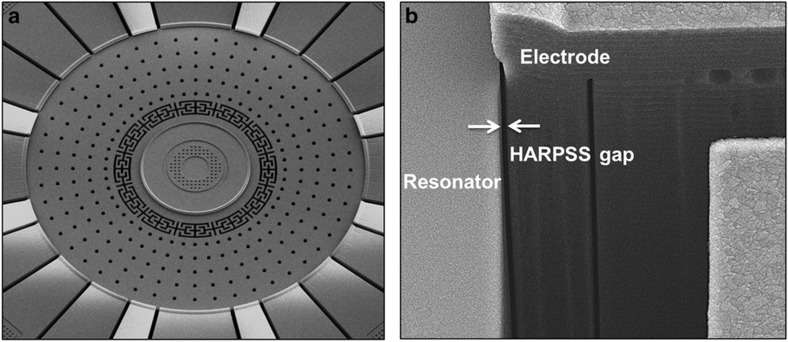
SEM view of the SD-BAW gyroscope (**a**), and close-up view of the 270 nm capacitive gap implemented with the HARPSS™ process (**b**). BAW, bulk-acoustic wave; HARPSS, high aspect-ratio combined poly and single-crystal silicon; SD, substrate-decoupled.

Figure 7 shows an SEM view of the fabricated SD-BAW gyroscope. Devices were fabricated using a modified version of the high-aspect ratio poly- and single-crystal silicon (HARPSS™) process flow^7^, in combination with planar *X*/*Y*-axis gyroscopes^34^ and tri-axial accelerometers^35^ as part of a 6-degree-of-freedom system.

**Figure 8:**
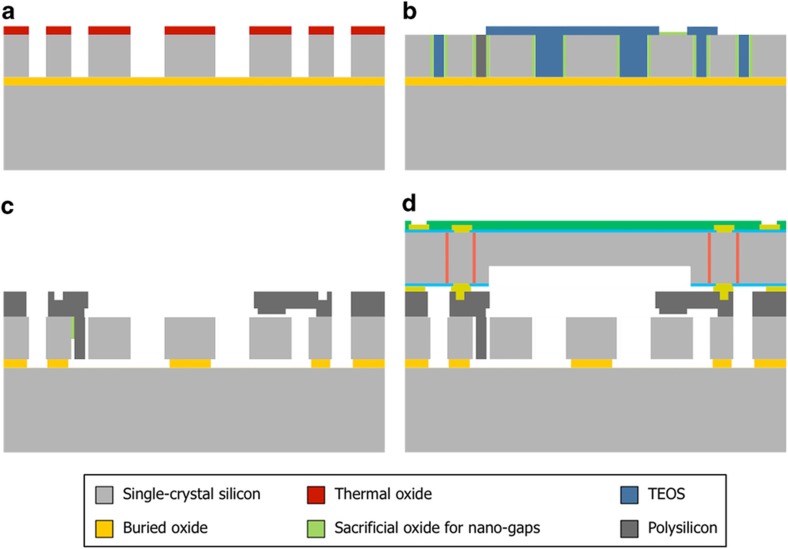
Modified process flow for the creation of SD-BAW *Z*-axis gyroscopes in conjunction with planar *X*/*Y* gyros and tri-axial accelerometers. (**a**) Structure is defined in SOI substrate via DRIE. (**b**) Capacitive gaps are defined by thermal oxidation, and trenches are refilled with polysilicon or TEOS to create electrodes. (**c**) Structure is released, and (**d**) device wafer is capped at a pressure of 1 to 10 Torr. BAW, bulk-acoustic wave; SD, substrate-decoupled.

The process uses SOI wafers with a 40-μm-thick structural layer and a 2-μm-thick buried oxide. Lateral trenches are etched via DRIE on the device layer using a thermal-oxide mask to outline the resonator features and the surrounding electrodes (Figure 8a). A 270-nm-thick layer of sacrificial oxide is then grown to define the lateral (in-plane) capacitive gaps. Next, the trenches adjacent to the electrodes are re-filled with polysilicon; all other trenches are refilled with sacrificial TEOS. A second layer of sacrificial oxide (300 nm thick) is grown to define the out-of-plane capacitive gaps used for the planar gyros and accelerometers (Figure 8b). This step is followed by the deposition and patterning of polysilicon that defines the vertical electrodes. The devices are then fully released in hydrofluoric acid (HF) (Figure 8c). Lastly, a capping wafer, which is processed independently, is bonded to the base wafer to provide hermetic wafer-level packaging (WLP) at a pressure level between 1 and 10 Torr (Figure 8d). Through-silicon vias (TSVs) provide electrical connections from the device electrodes to the top of the cap wafer, and metal traces route the signals to pins at the edge of the die to facilitate wire bonding with interface electronics.

## Characterization

### Standalone sensor characterization

**Figure 9:**
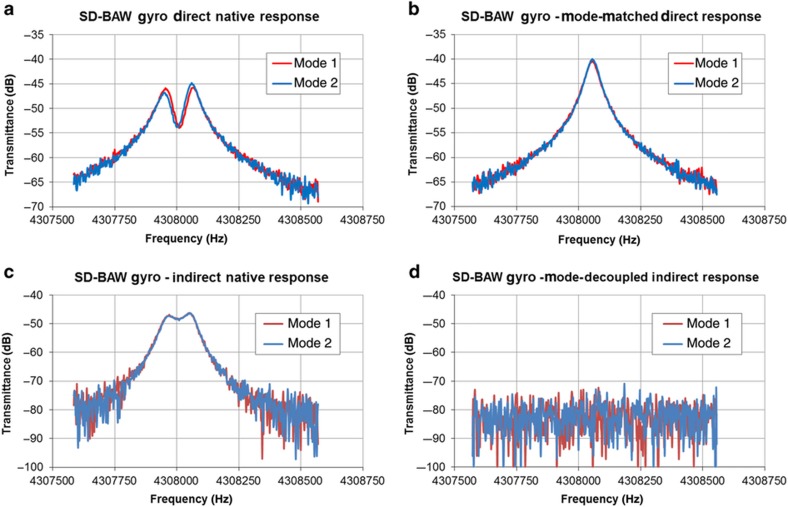
Frequency response of SD-BAW gyroscope at pickoff electrodes aligned directly (**a**, **c**) and orthogonal (**b**, **d**) to the mode being excited. Plots on the left are for the native response of the device, plots on the right are measured after frequency tuning and mode decoupling have occurred. BAW, bulk-acoustic wave; SD, substrate-decoupled.

WLP SD-BAW gyroscopes were first characterized as standalone devices in an open-loop configuration using a network analyzer. Figure 9a shows the typical frequency response read at the electrodes aligned with the drive mode (red trace) and the sense mode (blue trace) when each mode is individually excited. The native frequency split of 110 Hz can be reduced down to 0 Hz (Figure 9b) by adjusting the tuning voltage of the mode with the lower resonance frequency. For this particular sample, *V*_T1_=3 V is sufficient to match the frequencies with a resonator biased at *V*_P_=18 V and all other electrodes at 0 V (Figure 9b). Conversely, Figure 9c shows the signal response at the electrodes aligned with the opposite mode to that being excited. The magnitude of this signal is an indication of the level of mode-to-mode coupling caused by anisoinertia and anisoelasticity. By adjusting *V*_Q1_ to 2.5 V, the modes are fully decoupled (Figure 9d), showing no residual errors from damping coupling.

**Figure 10:**
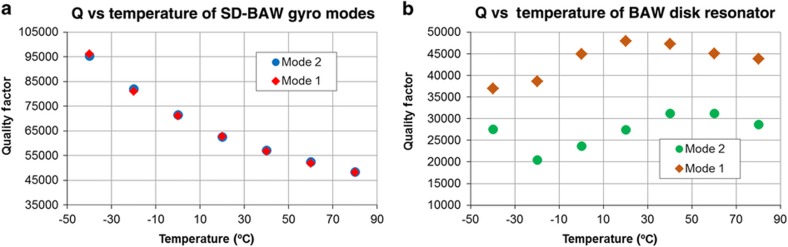
Quality factor variation over temperature of the second elliptical (*n*=3) modes of an SD-BAW gyroscope (**a**) and a center-supported BAW disk resonator implemented on a (100) SCS substrate (**b**). BAW, bulk-acoustic wave; SCS, single-crystal silicon; SD, substrate-decoupled.

The mode-matched *Q* value of the WLP SD-BAW gyro is near 62 000, which is similar to the predicted *Q*_TOTAL_ value of 68 000 (Table 1). To verify the effectiveness of the decoupling structure, the *Q* values of the second-elliptical modes of the gyroscope were monitored as the temperature was varied and were compared with those of a BAW disk resonator with poor substrate isolation (that is, its total *Q* is dominated by *Q*_anchor_). Figure 10a clearly shows that for the SD-BAW gyro, *Q* is well behaved as the temperature changes, showing the 1/*T*^*n*^ response expected by the combination of *Q*_TED_ and *Q*_SFD_ (in this particular case, *n*=1.65)^36^. Conversely, the *Q* values of the degenerate modes of a (100) SCS BAW disk resonator behave erratically over temperature, showing that the losses are highly dependent on the boundary conditions and properties of the substrate and how these vary with temperature (Figure 10b). Even more importantly, unlike the BAW disk resonator, the *Q* values for both modes of the SD-BAW gyro change identically with temperature, which guarantees that there will be no environmentally dependent damping coupling.

### System-level characterization

SD-BAW gyroscopes were interfaced with an application-specific integrated circuit (ASIC) to perform a full system-level characterization of the device. The ASIC consists of a drive channel that establishes an amplitude-regulated self-oscillation loop around the drive mode and a sense channel with low-noise front-end electronics. These components are followed by an I/Q demodulator that extracts the rotation-rate signal from its carrier and monitors the quadrature level. The quadrature signal is compared with a reference signal and is then fed back onto the mode-decoupling electrodes to maintain the gyro at an optimal operation state^37^. The ASIC is also equipped with a charge pump to generate the polarization and tuning voltages necessary to bias the sensor.

**Figure 11:**
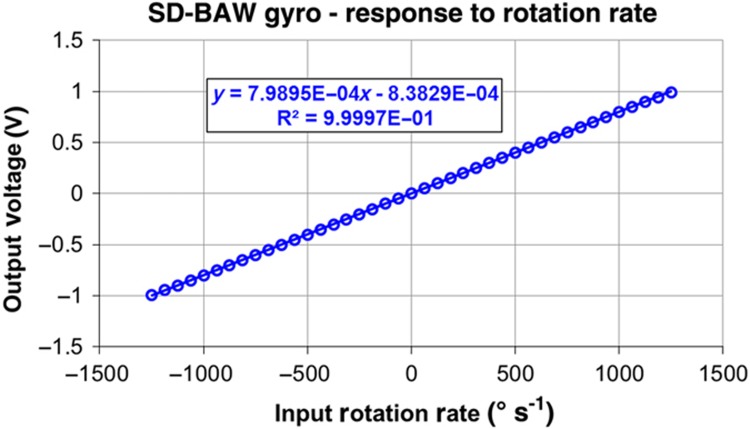
Output response of SD-BAW gyroscope to rotation-rate stimuli in increments of 62.5° s^−1^ and up to ±1250° s^−1^. The measured non-linearity in this range is 0.07%, which is limited by the interface electronics. BAW, bulk-acoustic wave; SD, substrate-decoupled.

The electronic gain of the sense channel was programmed to attain an overall scale factor of 800 μV (° s^−1^)^−1^, which corresponds to a full-scale range of ±1250° s^−1^ (Figure 11). The non-linearity of the part is smaller than 0.07% and is limited by the linear range of the output buffer.

**Figure 12:**
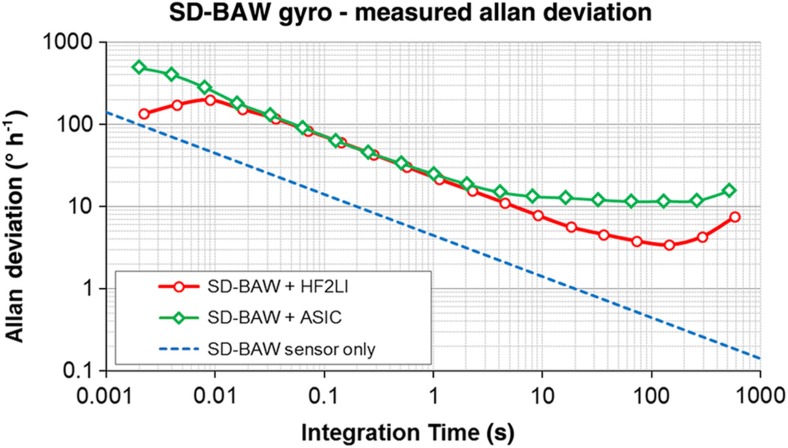
Allan deviation plot for SD-BAW gyroscope interfaced with customized ASIC and external electronics. The dotted trace shows the Brownian noise limit of the gyro sensor. ASIC, application-specific integrated circuit; BAW, bulk-acoustic wave; SD, substrate-decoupled.

Figure 12 shows the Allan deviation plot of the SD-BAW gyroscope. The complete sensor and ASIC system exhibits an angle-random walk (ARW) of 0.39°/√h (0.0065° s^−1^ (√Hz)^−1^) and a bias instability (BI) of 10.5°h^−1^, which are dominated by the thermal and 1/*f* noise of the ASIC, respectively. SD-BAW devices were also interfaced with JFET-input front-end discrete electronics with reduced 1/*f* noise and were demodulated using an HF2LI lock-in amplifier from Zurich Instruments (Zurich, Switzerland). With this configuration, the devices yielded BI values as low as 3.5°h^−1^, which are limited by a temperature-dependent feed-through (FT) signal coupling from the drive loop to the sense channel. Further improvements of the electronics thermal noise can reduce the ARW to its theoretical limit of 0.074°/√h (dotted trace in Figure 12), and a simple reorganization of the pin-out to minimize the FT can reduce the BI value of the system by at least 10 times.

**Figure 13:**
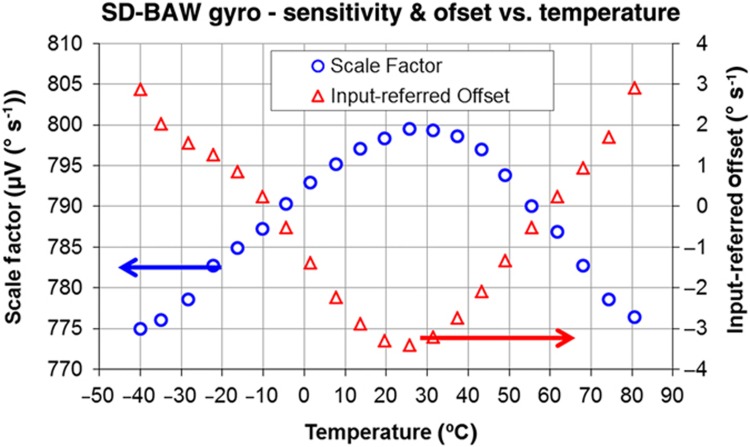
Variation of the scale factor and offset of an SD-BAW gyroscope as temperature varies. BAW, bulk-acoustic wave; SD, substrate-decoupled.

The temperature performance of the SD-BAW gyroscope was evaluated from −40 to 85 °C. The scale factor and offset change in a typical part are shown in Figure 13. A residual change in sensitivity of ±2.9% across the entire range was observed after applying a linear compensation slope to correct for the variation in *Q* described in Figure 10a. These higher-order residuals appear due to the 1/*T*^1.65^ temperature dependency of *Q* described in the previous section. However, because the response is quadratic and predictable, the use of a second-order compensation scheme can reduce the scale-factor variation across the entire temperature range to less than ±0.25%.

Conversely, the total change in the offset equals ±4° s^−1^ across the entire temperature range and is caused by the residual temperature-dependent FT signal coupling from the drive loop to the sense channel. The quadrature control loop, which suppresses any quadrature error signal, independent of its source, will introduce an error signal in the mode-decoupling electrodes when the FT level becomes too large. This error will lead to an unwanted change in the offset. Back-end quadratic compensation could decrease this variation to ±1° s^−1^, but the overall effect could be markedly reduced by a simple reconfiguration of the sensor pin-out to reduce the FT levels.

**Figure 14:**
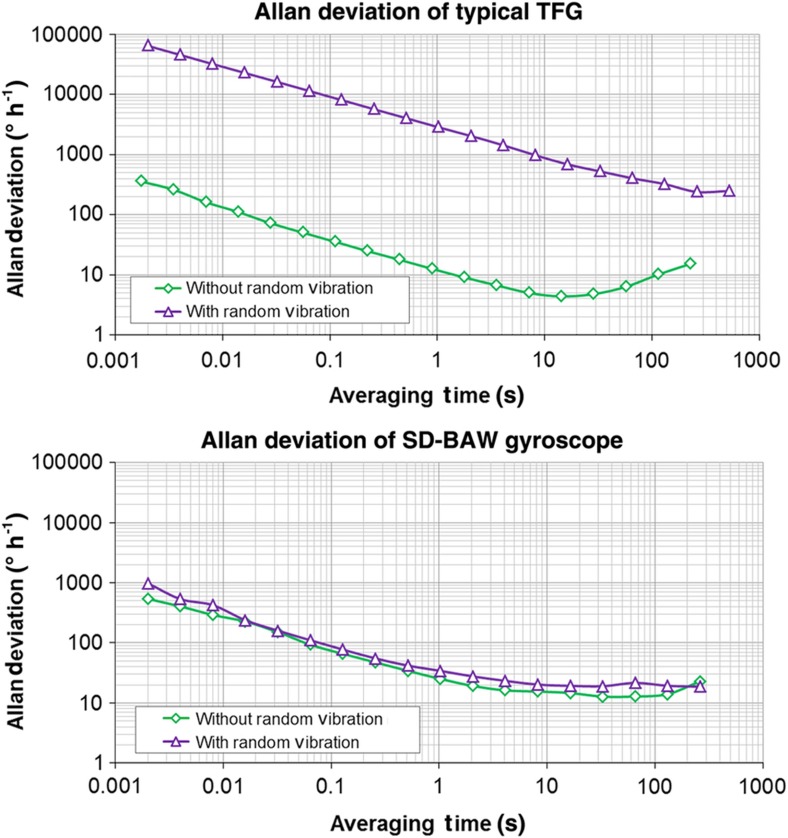
Change in Allan deviation response for an off-the-shelf TFG (top) and an SD-BAW gyroscope (bottom) in the presence of 14 g_RMS_ of random vibration with a white frequency spectrum from 50 Hz to 20 kHz. BAW, bulk-acoustic wave; SD, substrate-decoupled; TFG, tuning-fork gyroscope.

The robustness of the SD-BAW gyroscope with ASIC was verified by testing its immunity to random vibrations and shocks. The gyro was exposed to a 14-g_RMS_ random-vibration acceleration signal with a white frequency spectrum from 50 Hz to 20 kHz (1 g=9.8 m s^−1^). The measured vibration sensitivity along its most sensitive axis was as low as 0.012° s^−1^ g_RMS_^−1^, showing the advantage of the high-frequency nature of the gyro. Figure 14 shows a comparison of the Allan deviation with and without vibration for both a commercial off-the-shelf TFG (top) and an SD-BAW gyro (bottom). It is clearly shown that for the TFG, the noise performance degrades by several orders of magnitude, whereas for the SD-BAW gyro, a change in BI of less than 5° h^−1^ is observed. This result provides a clear indication of the superior noise performance of the SD-BAW gyro when operating in real environments.

**Figure 15:**
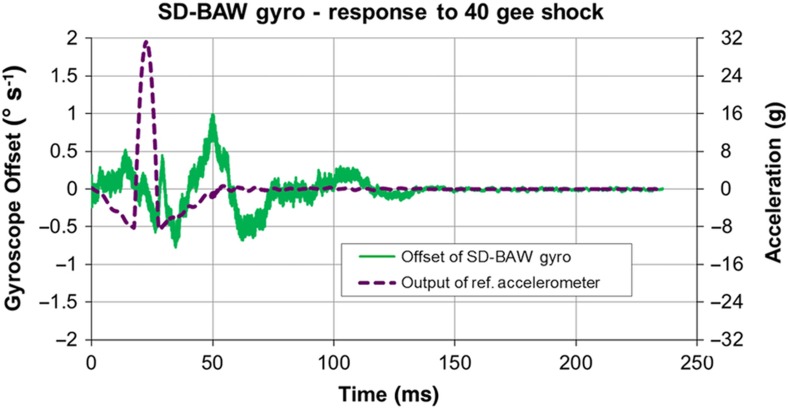
Maximum (peak) offset shift in the presence of a 40-g half-sine shock acceleration signal with a 10-ms duration. The dotted line represents the output of the reference accelerometer in the shock stage (right vertical axis); the continuous line represents the offset value of the gyro (left vertical axis).

The gyro system was also tested under a 40-g half-sine shock signal with a period of 10 ms along the most sensitive axis of the device. Figure 15 shows a maximum (that is, peak-to-peak) offset shift of only 1.75° s^−1^.

## Conclusion

This paper reported the design, simulation, fabrication, and characterization of a type of high-frequency mode-matched axis-symmetric gyroscope that exhibits superior environmental performance. A novel stress decoupling system is introduced to isolate the second elliptical modes of a BAW resonator from its substrate, reducing the effect of external sources of error on the sensitivity and the offset of the gyro. The current gyro performance is limited by the FT from the drive loop to the sense channel generated in the top metal traces that provide electrical connections to the electronics. Thus, further improvements in BI and variation of the offset and scale factor with temperature are expected through a simple reconfiguration of the pin-out. Table 2 highlights the performance metrics of the presented SD-BAW design.

## Figures and Tables

**Table 1:** Quality factor for second elliptical in-plane modes

Parameter	BAW disk resonator	SD-BAW gyroscope
Resonance frequency, *f*_0_	7.2 MHz	4.3 MHz
Squeeze-film damping, *Q*_SFD_	15 000 000 (1 Torr) 1 500 000 (10 Torr)	11 000 000 (1 Torr) 900 000 (10 Torr)
Thermoelastic damping, *Q*_TED_	150 000	74 000
Anchor loss, *Q*_anchor_	78 000	750 000 000
Total loss @ 10 Torr, *Q*_TOTAL_	49 000	68 000

BAW, bulk-acoustic wave; SD, substrate-decoupled; TED, thermoelastic damping.

**Table 2:** Performance metrics of the SD-BAW gyroscope

Parameter	Value	Units
Scale factor	800	μV (°s^−1^)^−1^
Full-scale range	±1250	°s^−1^
Non-linearity	0.07	%
Angle random walk	0.39	°/√h
Bias instability	10.5 (with ASIC) 3.5 (external electronics)	°h^−1^
Offset over temperature	±26 (measured) ±6.7 (with linear slope)	°s^−1^
Random vibration sensitivity	0.012	°s^−1^ g_RMS_^−1^
Max. offset at 40 g shock	1.75	°s^−1^

ASIC, application-specific integrated circuit; BAW, bulk-acoustic wave; SD, substrate-decoupled.
